# Teenagers Tell Better Stories After Improvisational Theater Courses

**DOI:** 10.3389/fpsyg.2021.638932

**Published:** 2021-03-16

**Authors:** Manon Blonde, Frédérique Mortelier, Béatrice Bourdin, Mathieu Hainselin

**Affiliations:** ^1^Université de Picardie Jules Verne, Département d'Orthophonie, Amiens, France; ^2^CRP-CPO, UR UPJV 7273, Université de Picardie Jules Verne, Amiens, France; ^3^Université de Picardie Jules Verne, Département de Psychologie, Amiens, France

**Keywords:** improv, language, education, skills, learning, story

## Abstract

Improvisational theater (improv) is a booming theatrical practice, applying in many fields (teaching, medicine or entrepreneurship). Its effects on cognitive and behavioral processes are beginning to be demonstrated, despite scientific publications that are still rare, particularly about language. This study aimed to evaluate the effects of improv on adolescent narrative skills. Twenty-seven middle school students were recruited and divided into two groups: an IMPRO group (*n* = 13), composed of novice and intermediate improvisers, and a CONTROL group (*n* = 14), composed of middle school students doing theater. The evaluation took place in two experimental times spaces 3 months apart (11 sessions). It consisted in the creation of a written narrative resulting from previously observed images. We used quantitative criteria to carry out the analysis of this story (coherence, cohesion, lexicon, and syntax), according to the methodologies of PELEA and EVALEO. We showed an effect of the improv on coherence, which suggests that the practice of improv introduces teenagers to improve the narrative skills and the planification of a story, unlike written theater.

## Introduction

### Improv

Improvisational theater (improv) is an art in which people go on stage without any scenario. They have to tell a story they didn't know themselves the minute before. Improv is a popular artistic practice which shares core values with teaching: establishing a safe environment, expanding our comfort zone to learn new skills and knowledge, listening to each other and working toward a common goal. The French Education Ministry even endorsed improv as a learning tool (Eduscol, 2017), consistent with recent projects involving teenagers, teachers, academics and students (Bernstein, 2014; Rossing and Hoffmann-Longtin, 2016; Hainselin et al., 2017; Felsman et al., 2019, 2020; Gao et al., 2019).

While long form performances are very common around the world, French-speaking countries (e.g., France and Canadian Québec) have a tradition of short form shows. The *match d'improvisation* (improvisational game) is a show with two 6-player teams, a referee and a master of ceremony. The audience votes after each scene for its favorite team (Chauvin, 2015). Although the *match d'improvisation* can include drama or musical improv, comedy is the most popular genre.

Improv is supposed to involve communication, flexibility, memory, language, creativity, problem solving, and co-construction (Bermant, 2013). Although teachers, learners and many qualitative studies reported these supposed benefits of improv (Sawyer, 2004, 2011; Landert, 2021), little scientific evidence using quantitative methods support these supposed benefits.

### Impact of Improv

The emotional impact of improv was only recently assessed. It appears that improv can help reduce anxiety and depression, as shown by Krueger et al. (2019) in 32 middle-aged adult patients. Recent publications, using an ecological paradigm (pre and post improv program assessment), highlighted well-being improvement in adults (Schwenke et al., 2020), social anxiety decrease in adolescents (Felsman et al., 2019) and, by using an experimental paradigm, improves uncertainty tolerance and affective well-being in young adults (Felsman et al., 2020).

Most improv research has focused on creativity and has found higher levels of creativity in teenagers and adults after improv practice (Lewis and Lovatt, 2013; Hainselin et al., 2018; Felsman et al., 2020), both in ecological and laboratory research. Control groups were very heterogeneous, with waiting list, improv games adapted to exclude uncertainty and co-construction, sports or sometimes without any control group at all. However, we didn't find quantitative study comparing improv with written (non-improv) theater. Memory was assessed in a single study and showed better ability to remember a text when played in an improvisation scene condition compared to reading only, or writing about the scene or group discussions conditions (Scott et al., 2001). This paper was the first to include language, with memory, as the focus of a quantitative research on improv.

### Language and Narrative Skills

Language is posited to be involved in improv, and some teachers use it within an artistic framework (López-González, 2015), to learn a second language (Matthias, 2009; Cocton, 2015) or improve the native language (Adebiyi and Adelabu, 2013). While most teachers and improv facilitators think improv can improve language, very little quantitative study to confirm this claim. In an 8-participant study without a control group, lexical skills were improved after a 4-week improv courses (Saygili and Saygili, 2016). However, while theater and improv need to tell a story, no study investigated this area.

Narrative skills allow talking about a topic or an event within a time and space framework, using a specific language. Narrative models (Fayol, 2000) describe different steps, as the classic hero journey, connected with the logical link and causation, to maintain a general cohesion. These narrative skills, learned in young teen years for early middle-school learning, are needed at school and for human relationships (Pesco and Gagné, 2017).

Thus, the aim of this preliminary study is to assess narrative skills evolution after improv courses compared to written theater courses. Considering the popularity of improv in middle schools and adolescence is a critical period for cognitive development, we focus our research on this population.

## Methods

### Population

Twenty seven teenagers were included in this study. We included 13 participants (11 females and 2 males) in the improv group and 14 participants (13 females and 1 male) in the control group (written theater). There was no significant age differences (improv group mean = 11.61; SD = 1.12; control group mean = 11.71; SD = 0.99) between the two groups. All were native French speakers. All teenagers gave their written consent to be part of the research and we had approval from their parents, headmasters and teachers. None of them had neurological or psychiatric history, visual, auditive nor writing disorders.

### Material

We used the EVALEO 6–15 Written Story subtest (Maeder et al., 2018) to assess narrative skills. EVALEO includes 9 pictures and is a quantitative assessment. We used its coherence assessment (number of indicators) because it is more detailed than the PELEA's (only yes/no answer).

To assess story structure and linguistic units, we used the PELEA (*Protocole d'Evaluation du Langage Elaboré de l'Adolescent*, teenager elaborated language assessment) Story subtest evaluation grid, assessing oral story structure and linguistic units (Guillon and Boutard, 2010). PELEA includes 3 black and white pictures and assess coherence, vocabulary richness, and syntax. These qualitative indicators were used and transformed into quantitative scores. We choose the PELEA pictures rather than EVALEO's ones to avoid any memory overload, common in daily speech therapist practice for this task, and the EVALEO grid to have a quantitative analysis (see Supplementary Material for assessment grids with an example).

EVALEO and PELEA are the usual speech therapist narrative skills assessment. For French-speaking teenagers, we only have these two tasks available. Combined together, these two grids allowed us to assess Number of words, Coherence (story structure, taking implicit into account), Cohesion (logic connectors, temporal clues, and adverbs), Vocabulary Richness (poor = 0, average = 2, and rich =5), and Syntax (very low = 0, low = 2, average = 4, and good = 6).

Participants had to write a story inspired by the three black and white PELEA pictures, with unlimited time and without orthographic errors. This assessment and analysis, including different factors, helped us to have an overview of the narrative skills.

### Design

We assess narrative skills just before the first improv course (T1) and within 1 week after the last course (T2), on the same week day and at the same time as T1. Considering the 11 weekly courses, T1 and T2 assessments were 3 months apart. Improv course content was like a previous work on improv in middle school (Hainselin et al., 2018) and are detailed in Table 1.

**Table 1:** Improv sessions description.

**Session number**	**Topic**	**Example of improv games**
Session 1	Presentation, basic improv games	Zip Zap Zop: people pass the energy across the circle (in the form of a Zip, a Zap, or a Zop), they make eye contact with the person they send the energy to
Session 2	Focusing	Within a circle, pass multiple imaginary balls of color without losing them or change any color.
Session 3	Listening	Walking with the eyes closed, only guided by another improviser's voice
Session 4	Reactivity	All participants had to rank themselves regarding different criteria (i.e. hair length) as quickly as possible
Session 5	Body language	Walk with a specific pattern and mimic the others' ones to observe and feel how it changes your perception.
Session 6	Emotions	Turning the emotion volume from 1 (a bit annoyed) to 5 (biggest anger ever)
Session 7	Co-construction	Yes, and: listening to one other improviser's idea and add a detail to make this idea richer
Session 8	Location	Everyone takes a position with a specific shape to make the group look like a specific place (e.g., a zoo)
Session 9	Language and voice	Speak gibberish to sell an imaginary object in 90 s
Session 10	Storytelling	Building a whole story from scratch, with some clue from the facilitator
Session 11	Improv	Playing a story only with a topic 10 s before starting

Written theater courses were classic courses, studying modern texts, avoiding a gap of language between the two groups. Participants had to work on texts, learn and play it with gestures. They had feedback on the speaking performances and way to move their body into space as in the improv courses.

### Statistical Methodology

All data were analyzed with JASP (Love et al., 2019). We carried out each statistical analysis using repeated measured ANOVA with Group (Improv, Control) as between participants' factor and Condition (T1, T2) as the within-participant factor. For Vocabulary Richness and Syntax, given the absence of homogeneity, we ran Wilcoxon analysis.

The raw data and JASP analysis file are available on the Open Science Framework (OSF) platform at https://osf.io/kqjeb/.

## Results

All scores mean and standard deviation are in Table 2.

**Table 2:** Means and standard deviation for each condition and group.

	**Improv**		**Theater**	
	**T1**	**T2**	**T1**	**T2**
Number of words: Meand (SD)	196 (63)	208 (85)	230 (77)	249 (76)
Coherence: Meand (SD)	23 (4)	27 (5)	21 (3)	19 (3)
Cohesion: Meand (SD)	41.10 (5.70)	45.57 (4.40)	40.06 (7.69)	41.56 (4.50)
Vocabulary Richness: Meand (SD)	3 (2)	4 (1)	3 (1)	3 (1)
Syntax: Meand (SD)	4 (1)	5 (1)	4 (2)	4 (1)

### Number of Words

There was no main effect of Group [*F*_(1)_ = 2.078, *p* = 0.162], Condition [*F*_(1)_ = 1.234, *p* = 0.277] nor Group × Condition interaction [*F*_(1)_ = 0.078, *p* = 0.782].

### Coherence

**Figure 1:**
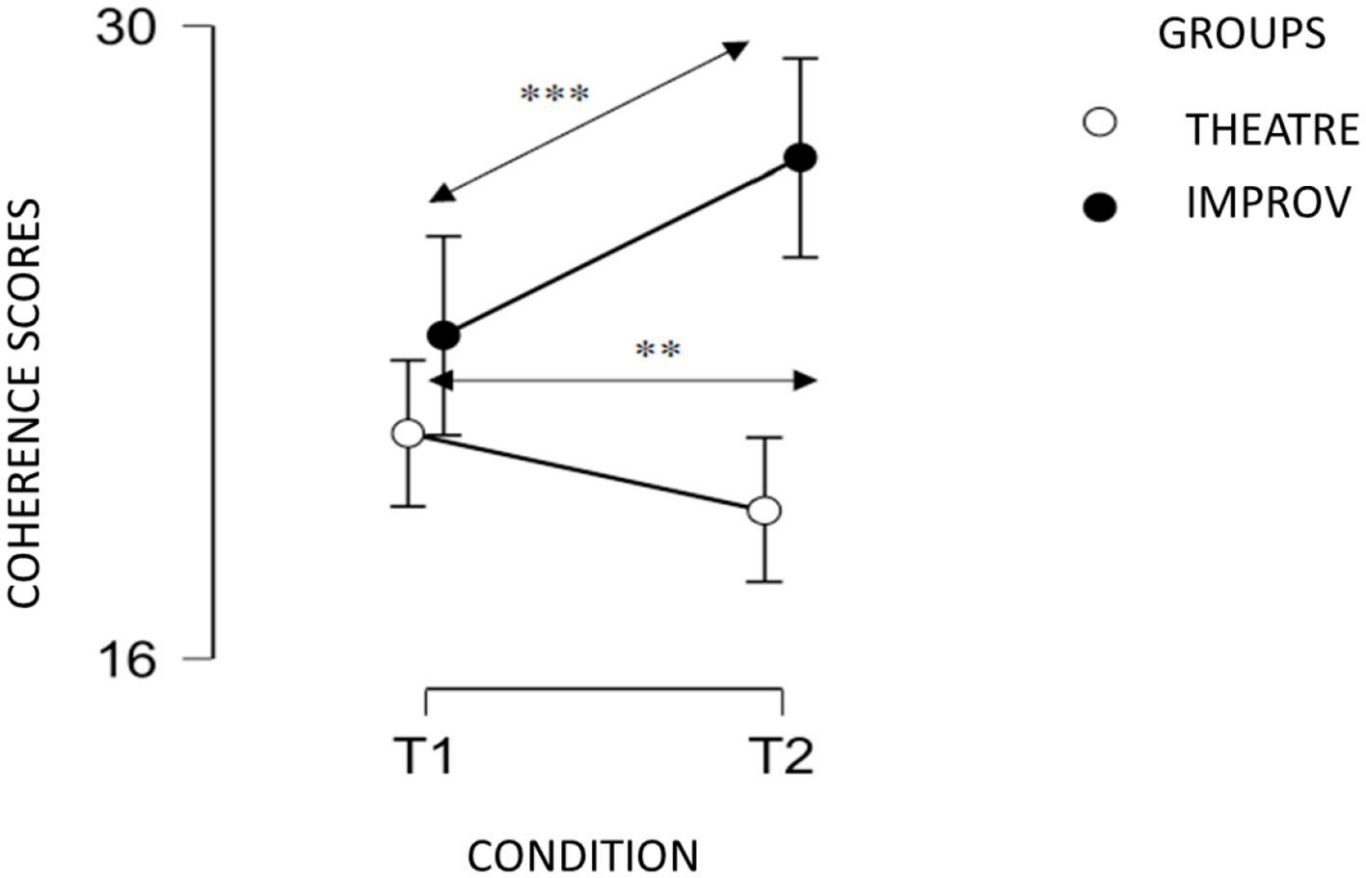
Coherence scores at T1 and T2 for improv and theater groups. **p < 0.01; ***p < 0.001.

We found a main effect of Group [*F*_(1)_ = 18.745, *p* < 0.001] and a Group × Condition interaction [*F*_(1)_ = 10.264, *p* = 0.004], such as T2 scores were higher than T1 scores for the improv group (see Figure 1). There was no Condition effect [*F*_(1)_ = 1.576, *p* = 0.221].

### Cohesion

There was no main effect of Group [*F*_(1)_ = 3.597, *p* = 0.069], Condition [*F*_(1)_ = 2.612, *p* = 0.119] nor Group × Condition interaction [*F*_(1)_ = 0.889, *p* = 0.355].

### Vocabulary Richness

**Figure 2:**
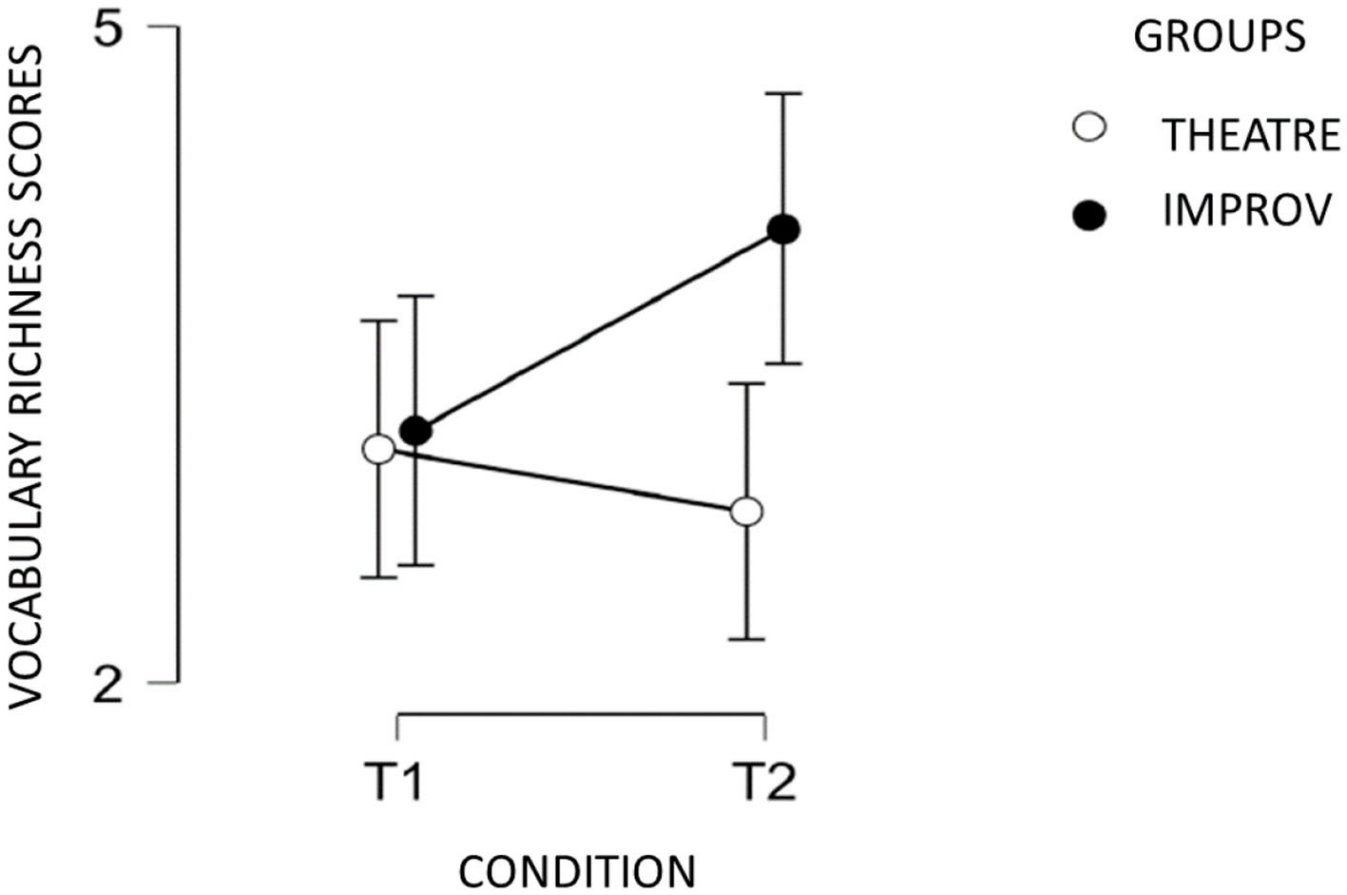
Vocabulary Richness scores at T1 and T2 for improv and theater groups.

The Wilcoxon analysis didn't show a T1/T2 improvement for the theater group [*W* = 7, *p* = 0.071] nor the improv group, although there was a trend to significance [*W* = 0, *p* = 0.072] (see Figure 2).

### Syntax

The Wilcoxon analysis didn't show a T1/T2 improvement for the theater group [*W* = 17.5, *p* = 0.149] nor the improv group [*W* = 17.5, *p* = 0.129].

## Discussion

Our preliminary results confirm than improv can help enhance narrative skills, for Coherence and with a trend to significance for Vocabulary Richness. Improv could facilitate the translation process and, more specifically, the structuration of the text by increasing the relationship between the statements, but not the cohesion. The results could suggest that improv courses made it easier to access lexicon. By contrast, improve courses has no effect on the length of text.

The “yes, and” rule in improv could be a way to build a coherent story together. By doing so, improvisers have to connect each other's story, and give the audience a coherent story, which could be an explanation of the coherence score improvement.

During human interaction, we can trigger vocabulary we don't use spontaneously. In a conversation, we have more chance to use a syntactic structure, as a passive voice, after someone's used it (Bock, 1986). This effect was also observed in young children (Shimpi et al., 2007; Bourdin et al., 2018), in aphasic patients (Hartsuiker and Kolk, 1998) or in children with language disorders (Coco et al., 2012; Bourdin and Leuwers, 2020). Linguistic input influences language production (Branigan and McLean, 2016).

Improvisers have to use other one's vocabulary to keep the story coherent. For example, if the first sentence is about the hippocampus and memory encoding, all improvisers will probably have to use “hippocampus” and “memory encoding at some point. Although they don't use it, they will listen to it multiple times.

As well, produce a text from a series of items semantically related (items related to a single script (related) help writers to produce a coherent sequence (Bourdin and Fayol, 2002; Bourdin et al., in review). Future research could focus on these specific topics with specifically designed improv exercises (“yes, and…”) and scientific paradigm (short task with only one exercise).

This research is the first quantitative experiment to assess narrative skills and to compare improv to (written) theater. Further research is needed to assess the various components more accurately involved in production.

### Future Direction for Research

More participants are needed to confirm these preliminary results, and future research could focus on the cognitive profile of the participants. Beyond a *g* factor, the assessment of planification and memory performances, supposed to be involved in narrative skills, could help us to have a better understanding of the improv impact on language (Losh and Capps, 2003). The use of qualitative methods, besides a quantitative one, could be of great interest to assess the singularity of improvisers.

The need to start stories from nothing but one's own imagination, instead of a text in theater, could be the reason for the difference between our two groups. The need for co-construction and the better confidence to speak in public (Felsman et al., 2020) could also be helping factors. Further research might focus on individual vs. group exercises with a specific design.

This research took place in an ecological context, with classic improv courses, as in multiple recent publications (Hainselin et al., 2018; Felsman et al., 2019; Schwenke et al., 2020). On the one hand, ecological research involving an 11-week improv program is a great way to assess the impact of classic improv training, but it is difficult to know the specific impact of improv because of many parasite variables. Another study had a laboratory context (i.e., one session with 3–5 exercises within lab rather than school or theater) to assess improv (Lewis and Lovatt, 2013; Felsman et al., 2020) and find improvements after one short improv workshop (30 min). This latter paradigm is, however, less likely to be implemented in weekly routine and are supposed to have less impact over time. Instead of set the two approaches against each other, future research might combine the two approaches for the same variables (narrative skills, divergent thinking, well-being…) and assess their long-term impact with follow-up months after the end of the improv courses.

### Applied Improvisation

Beyond the theater, we can find a developing interest for applied improvisation (i.e., improv with a different aim than artistic performance) by professionals: managers (Dohe and Pappas, 2017), negotiators (Balachandra et al., 2005), scientists (Bernstein, 2014) and teachers (Rossing and Hoffmann-Longtin, 2016), medical doctors (Fu, 2019; Gao et al., 2019) and psychologists (Hainselin et al., in press, DeWever et al., in revision) took part into improv workshops, in order to use improv tools and values in their respective professional context.

For speech therapists, applied improvisation can help to be more creative, more ready to deal with uncertainty. Speech therapists already use (fun) exercises and adapt existing ones, links with different ideas to bring them where the therapists want. It helps to know how to link information together, be open to new ways to bring two different worlds together (therapist and patient), beyond the classic desk/chair situation.

Considering our preliminary results, speech therapists could use applied improv within their clinical setting to help teenagers to deal with narrative difficulties. More broadly, they might work with teachers and parents to use improv exercises in different contexts, including outside hospitals and private practice. For example, try different ways to speak with different body positions to know how the voice change in this context. Play short scenes and embody characters, animals or objects (i.e., embody a lion to help mental representation, physical characteristics, and associate it with words). For young patients (children and teenagers), it can help them to improve their self-esteem and their commitment in the therapy process. By doing so, it would be a great opportunity to bring linguistics skills to many more children and teenagers. People with language impairments could also benefit from a better inclusion through fun games, and from language-centered improv exercises without facing the discomfort to leave the classroom or going to the health professionals' office.

## Data Availability Statement

The original contributions presented in the study are included in the article/Supplementary Material, further inquiries can be directed to the corresponding author/s.

## Ethics Statement

Ethical review and approval was not required for the study on human participants in accordance with the local legislation and institutional requirements. Written informed consent to participate in this study was provided by the participants' legal guardian/next of kin.

## Author Contributions

MB and MH wrote the manuscript. MB includes participants. MB and MH ran statistical analysis. FM and BB drafted the manuscript. All authors contributed to the article and approved the submitted version.

## Conflict of Interest

The authors declare that the research was conducted in the absence of any commercial or financial relationships that could be construed as a potential conflict of interest.
