# Supplementary material for: Teenagers Tell Better Stories After Improvisational Theater Courses
Source: Front Psychol. 2021 Mar 16;12:638932. doi: 10.3389/fpsyg.2021.638932 (PMC8008055; doi:10.3389/fpsyg.2021.638932)
Supplement: Supplementary file 1 [file Table_1.pdf]

Supplementary material. Example of a story

Il était une fois un roi qui demeurait dans un château perché sur une colline. Une nuit, il dormait paisiblement, quand soudain, des éclairs foudroyèrent le château, ce qui réveilla le roi. Affolé, il courut hors du château quand il vit sortir des éclairs un chevalier porté par un majestueux cheval blanc doté d'ailes. Au même moment, le roi se fit frapper par la foudre. Le chevalier vola à son secours mais il fut trop tard. Le roi mourut dans ses bras car le chevalier n'arriva pas à le sauver. Il l'enterra donc et planta son épée sur sa tombe en signe de condoléances. Depuis ce jour, à chaque nuit de tempête, on peut apercevoir un chevalier qui vole sur le dos d'un majestueux cheval blanc doté d'ailes autour des ruines du château.

Analysis:

Verbal inflexion (15) : était, demeurait, dormait, foudroyèrent, réveilla, courut, vit, se fit, vola, fut, mourut, arriva, enterra, planta, peut.

Subject personal pronoun (5) : il, il, il, il, on.

Complementary personal pronoun (2) : le, l'.

Relative pronoun (2) : qui, qui.

Possessive or demonstrative adjective (5) : son, ses, son, sa, ce.

Lexicla coreferent (11) :

- un roi, le roi, il, le, l'
- un château, le château
- un chevalier, le chevalier, il

Relative group (2) : qui demeurait dans un château… qui vole sur le dos…

Completive group (1) : ce qui réveilla le roi.

Logical connectors (5) : par, par, mais, car, donc.

Temporal mark (2) : quand soudain, quand.

Adverbs (2) : paisiblement, trop.

Supplementary Material. PELEA and EVALEO assessment grids.

**Story structure**	**SCORES**
1. Announcement	2
Initial boundary process *(il était une fois)*	2
2. Indications	8
Who? (un roi)	2
When ? (une nuit)	2
Where? (dans un château perché sur la colline)	2
What? (éclairs qui foudroient le château)	2
How? (N/A)	0 .
3. Triggeran	2
Rivalry (N/A)	0
Attack (N/A)	0
Old man or princess death (le roi meurt)	2
Revenge (N/A)	0
5. Conclusion	2
Outcome (hommages/condoléances)	2
6. Ending	0
Final boundary process (N/A)	0
**Total story structure**	**14**

**COHERENCE**	**SCORES**
What are we talking about?	1
Link between sentences	1
Chronology	1
Permanence frame	1
Permanence of the characters	1
Taking implicit into account	4
**Total coherence**	**0**

**COHESION**	**SCORES**
1. Coreference	40
Verbal inflexion	15
Personnal pronoun	5
Personnal pronoun with direct complement	2
Relative pronoun	2
Other pronouns (possessive, demonstrative, interrogative, numerical…)	0
Possessive or demonstrative adjective	5

Lexical coreferent	11
2. Subordinate group	3
Relative group	2
Completive group	1
Circumstantial group	0
3. Logical connectors	5
Logical connectors (cause, goal, condition…)	5
4. Non verbal time mark	10
"Andt"	6
Time mark (temporal adverbs…)	2
Adverbs (except time mark)	2
Presentatives (this is, there is…)	0
**Total cohesion**	**58**

**VOCABULARY RICHNESS**	**SCORES**
Poor = 0	0
Average (1 or 2 elaborated words) = 2	0
Rich (more than 2 elaborated words) = 5	5
**Total vocabulary richness**	**5**

**SYNTAX**	**SCORES**
Bad (0)	0
Low (2)	0
Normal (4)	0
Good story, elaborated, easy to read (6)	6
**Total syntax**	**6**

Number of words	138
**RESULTS**	**SCORES**
Coherence	23
Cohesion	42.02
Vocabulary richness	5
Syntax	6
**TOTAL Story, including number of words**	**76.02**
